# Tidal volume accuracy during non-invasive ventilation with modern neonatal mechanical ventilators

**DOI:** 10.1186/cc14342

**Published:** 2015-03-16

**Authors:** K Moon, S Mizuguchi, K Tachibana, M Takeuchi

**Affiliations:** 1 Osaka Medical Center and Research Institute for Maternal and Child Health, Izumi City, Osaka, Japan

## Introduction

Maintaining the appropriate tidal volume (VT) is important for success of lung protective ventilation. However, in neonates, the presence of airway leaks may increase the errors in the delivery of tiny VT, which raises a concern of ventilator-induced lung injury. This study is to investigate the accuracy of VT delivery during non-invasive ventilation (NIV) with modern neonatal ventilators.

## Methods

Using a lung simulator for a patient body weight of 3 kg, we measured the actual delivered VT in the lung and compared it with the value displayed on the ventilator in six ventilators. We tested 18 conditions with various combinations of respiratory mechanics (normal, restrictive, obstructive), leak levels (0, 1.0, 1.5 l/minute), and PEEP settings (5, 10 cmH_2_O). All conditions were tested in NIV mode. The pressure level was set to achieve VT to the lung at 6 to 7 ml/kg. All other settings were: F_1_O_2_ 0.21, I_time_ 0.6 seconds, f 25/minute, and default rise time. We calculated the mean errors of the ventilator-displayed VT at various levels of airway leak.

## Results

The VT mean error values are presented in Table 1. When no leak existed, the mean error was less than 5% in all ventilators except one (C3) which showed a mean error of 26%. As the leak level increased, three ventilators (C3, G5, and VN500) showed marked differences between the delivered and displayed VT. In particular, the VN500 could not operate in the large leak condition. The other three ventilators (PB840, PB980, Servo i) showed acceptable VT accuracy across all conditions tested.

**Table 1:** 

Leak amount (l/minute)	Hamilton C3	Hamilton G5	Servo i	PB840	PB980	VN500
0	26 (15)	4 (6)	3 (2)	2 (7)	-4 (2)	2 (2)
1.0	-9 (5)	9 (5)	-2 (2)	-5 (4)	-2 (4)	20 (5)
1.5	16 (8)	-14 (8)	-2 (2)	-6 (4)	-2 (3)	33 (6)^a^

Data presented as mean (SD), %. ^a^Unable to measure in one case.

## Conclusion

Tidal volume accuracy during neonatal NIV varies greatly among different ventilators and leak conditions. This must be considered in neonatal ventilation management to avoid overventilation or underventilation.

